# Understanding training needs in eating disorders of graduating and new graduate dietitians in Australia: an online survey

**DOI:** 10.1186/s40337-021-00380-1

**Published:** 2021-02-18

**Authors:** Elyse Denman, Elizabeth Kumiko Parker, Mellisa Anne Ashley, Deanne Maree Harris, Mark Halaki, Victoria Flood, Anita Stefoska-Needham

**Affiliations:** 1 SMART Foods Centre, Illawarra Health and Medical Research Institute, School of Medicine, Faculty of Science, Medicine and Health, University of Wollongong, Wollongong, NSW 2522 Australia; 2 Department of Dietetics & Nutrition, Westmead Hospital, Westmead, NSW 2145 Australia; 3 Sydney School of Health Sciences, Faculty of Medicine and Health, The University of Sydney, Sydney, NSW 2006 Australia; 4 Adult Eating Disorder Service, Western Sydney Local Health District, Sydney, NSW 2145 Australia; 5 Department of Dietetics & Nutrition, Tamworth Rural Referral Hospital, Tamworth, NSW 2340 Australia; 6 Western Sydney Local Health District, Sydney, NSW 2145 Australia

**Keywords:** Eating disorders, Dietitian, Education and training

## Abstract

**Background:**

Following recent reforms by the Australian Government to the Medicare Benefits Schedule, people living with a diagnosed eating disorder (ED) in Australia have greater access to dietetic services. However, new graduate dietitians anecdotally lack confidence to provide appropriate interventions to support patients with an ED. Therefore, this cross-sectional study aims to explore the perceived confidence, and educational and professional development needs of student dietitians and new graduate dietitians in the area of EDs.

**Methods:**

An online survey with 17 questions was designed, consisting of a combination of discrete (yes/no) questions, free text, ordered scales and 5-point Likert scales. Student dietitians, and first- and second- year graduates (*n* = 1456) were approached via email as potential participants, from the professional organisation Dietitians Australia member list. Survey data was analysed using descriptive statistics and odds ratios.

**Results:**

In total, 150 surveys were completed, with a response rate of 10.3%. Respondents reported a lack of confidence in managing patients with an ED and implementing ED treatment approaches (81 and 95%, respectively). However, participants previously exposed to patients with an ED, such as anorexia nervosa, were 4.7 times (95% CI 1.72, 12.97) more likely to be confident compared to those not exposed to patients with an ED. The majority of respondents (37%) stated they would seek assistance from other dietitians, and develop their skills via online webinars (27%) and workshops (25%).

**Conclusions:**

This survey identified that final year dietetics students and new graduate dietitians perceive lower levels of confidence to practice in the area of EDs. The desire for further ED-specific training and education was reported.

## Plain English summary

Reforms by the Australian Government to the Medicare Benefits Schedule in 2019, have allowed greater access to dietetic services for people living with an eating disorder (ED). However, the confidence level of new graduate dietitians to treat patients with an ED has been reported as low. This study aimed to explore the perceived confidence, educational and professional development needs in the area of EDs, of students enrolled in their final year of an accredited nutrition and dietetics university program and dietitians who had graduated within the previous two-year period. Between 28th May 2019 and 25th June 2019, 1456 final year student and new graduate dietitians were invited to participate in an online survey, distributed by the professional organisation Dietitians Australia membership list. Of the 150 surveys completed, 81% of respondents reported a lack of confidence in managing patients with an ED and 95% reported a lack of confidence in using various ED treatment approaches. The desire for further eating disorder specific training and education was reported.

## Background

Eating Disorders (EDs) are psychiatric illnesses which are typically characterised by persistent disordered eating behaviours and/or compensatory behaviours to control body weight or shape [1]. EDs, including anorexia nervosa, bulimia nervosa, binge eating disorder and avoidant/restrictive food intake disorder (ARFID), are associated with severe psychological distress and, may also lead to complex long term medical comorbidities such as infertility, cardiovascular symptoms, digestive disorders, fatigue and pain [1–4]. Anorexia nervosa in particular, is the most fatal of any psychiatric disorder [5].

In Australia, prevalence data indicates approximately 16% of adults and 8–15% of adolescents are affected with an ED [6, 7]. With early diagnosis and appropriate interventions, including preventative health and wellbeing interventions, the incidence, prevalence and severity of EDs may be reduced [8, 9]. A recovery orientated approach to treatment is recommended, with coordinated continuous, multidisciplinary care from skilled professionals, including dietitians [8].

Accessibility to treatment has remained a concern for this patient group. It has been reported that 20–43% of people with EDs seek treatment for their ED from a healthcare provider [10–12], and health funding for ED management has been limited, with only 37 dedicated adult hospital beds available across Australia for EDs in 2016 [13]. For those seeking outpatient treatment, previously a maximum of 10 sessions for psychological services from a range of eligible treating health professionals and 5 dietetic consultations per year were funded by the Australian Government under the Medicare Benefits Schedule (MBS). This was deemed to be insufficient to treat patients with an ED to full recovery and resulted in large out-of-pocket treatment costs for those individuals [14]. In recognition of this shortfall in services, the New South Wales Health Department released a state-wide service plan for people with EDs in 2013 to implement strategies for improved primary and community based interventions, as well as local hospital interventions [15]. Additional reforms by the Australian Government to the MBS were introduced in November 2019, offering eligible patients diagnosed with an ED up to 40 sessions for psychological services from a range of eligible treating health professionals, and 20 dietetic sessions per year [16]. While the rate of uptake of the scheme is currently unknown, these reforms have the potential to reduce hospitalisation costs, increase recovery rates and improve the overall quality of life for many people.

These changes to the MBS for the treatment of EDs represent potential growth in employment opportunities for the dietetics profession, in particular for new graduates to work in private practice compared to more traditional hospital-based settings [17]. To this end, new graduate dietitians may be more likely to encounter both an undiagnosed patient with an ED or a patient with a diagnosed ED in private practice clinics.

Practising safely and within a defined scope of practice is essential for the delivery of safe care to ED patients, across all disciplines. From a dietetic perspective, an understanding of how to work within one’s scope of practice can be facilitated by adherence to the Dietitians Association (DA) Code of Professional Conduct [18] and the Ethical Practice Statement [19], and guidance from entry level dietetic competencies (*National Competency Standards for Dietitians* - NCSD) [20], which focus on general professional attributes (knowledge, attitudes and skills). Dietitians practising in the ED area may also refer to ED-specific national practice standards developed by the National Eating Disorder Collaboration (NEDC) for health care providers [21]. The Australia and New Zealand Academy for Eating Disorders (ANZAED) have recently published ED treatment principles and general clinical practice and training standards for mental health professionals and dietitians [22]. The ANZAED have also published practice and training standards specific for dietitians providing ED treatment, to ensure the safe and effective treatment of individuals with an ED [23]. Furthermore, in response to the lack of clinical practice guidelines for dietetic treatment of EDs, McMaster et al. (2020) [24] developed consensus-based guidelines for the outpatient setting.

Inadequate training in EDs has been identified as a workforce challenge across various health professionals, including dietitians [25–30]. Additionally, there are concerns that health professionals with limited clinical experience especially in the area of ED management principles, may unintentionally cause harm or even delay recovery [31]. Maguire et al. (2019) [32] report that practitioners’ poor self-belief to practice competently may create stigmatised attitudes towards patients with EDs and could impede the clinical care process. This in turn may result in an absence of or untimely referrals to specialist treatment services, such as dietetics [32]. Whether dietetic students and new graduate dietitians feel the same is currently unknown due to the paucity of literature in this area.

Therefore, the present study aims to explore the perceived confidence levels of student and new graduate dietitians to treat patients with an ED, their confidence in working with various ED treatment approaches, and their professional development and training needs. These findings may be used to inform the development of tertiary and post graduate training opportunities that enhance dietitians’ confidence to practice in the ED field, and in the longer-term, assist in achieving better outcomes for people with EDs through the provision of improved dietetic care.

## Methods

This cross sectional study used an exploratory research approach, utilising a purpose built online survey. Participants were recruited from the professional organisation DA membership list following their expression of interest to participate. Inclusion criteria were students enrolled in their final year of an Australian nutrition and dietetics university program and dietitians who had graduated within the previous two-year period. Engaging the DA membership list increased the likelihood of representativeness, as DA is the peak body for dietetic and nutrition professionals in Australia [33]. Participation in the study was anonymous and voluntary, with consent implied by online survey completion. Ethical approval was received from The University of Sydney Human Research Ethics Committee (Project No. 2019/078). This study was prepared based on strengthening the reporting of observational studies in epidemiology (STROBE) statement for cross-sectional studies [34].

The survey consisted of 17 questions, including a combination of discrete (yes/no) questions, free text, ordered scales and 5-point Likert scales. Participants were asked about their perceived confidence to treat patients with an ED and the use of different treatment modalities, seeking advice and their preferred methods for further training and education. The treatment approaches explored included dietary counselling [35, 36], mindful eating [37, 38], Health at Every Size (HAES) [39, 40], intuitive eating [41, 42], motivational interviewing [35, 36, 43], developing meal plans for weight restoration [35], and the Real Food Guide [44]. While HAES is not a recognised treatment approach specifically for ED treatment, it was included in the survey as the HAES weight inclusive approach to health has emerged in the ED field, in particular for individuals with binge eating disorder [40]. Face validity of the survey content was addressed in consultation with the professional organisation DA and a group of dietetic leaders in EDs, who also provided insight into commonly used treatment approaches used in dietetic practice for individuals with EDs. Prior to dissemination, the survey was piloted online with 5 independent dietitians for internal consistency and face validity. The survey was open for 4 weeks from the 28th May 2019.

Data collected was analysed in Microsoft Excel (2013). Descriptive statistics were generated for all reported measures, summarised in counts and percentages. The 5-point-Likert scale responses were dichotomised into binary responses such as ‘Agree’ vs ‘Not agree’ to show clear trends in the data. Specifically, ‘Strongly agree’ and ‘Agree’ responses were grouped as ‘Agree’. ‘Strongly disagree’, ‘Disagree’ and ‘Neither agree nor disagree’ were grouped as ‘Not Agree’. ‘Neither agree nor disagree’ was counted as ‘Not agree’ based on the assumption that those people were less likely to feel confident in a specific area. For some questions, the mean percentage of responses was calculated. In order to calculate odds of association between exposure and confidence, responses were cross-tabulated between those who had encountered a patient with an ED and those who had not. Responses were dichotomised; whereby, disagreement with the statement in all settings was counted as ‘not confident’, while agreement in at least one setting was counted as ‘confident’. Missing data was treated as follows; a participant with non-responses for a specific question was included in the study; however, the non-responses were accounted for and not included in the analysis of that individual question. As a result, the sample size varied for each question.

## Results

In total, 150 out of 1456 people participated in the study, yielding a 10.3% response rate. Participants comprised of primarily recent graduates (77%) and a smaller proportion of final year students (21%). Characteristics of participants, including their previous encounters with a patient with an ED, in either an inpatient or outpatient setting, are shown in Table 1.

**Table 1:** Characteristics of survey participants

	Responses n (%)
*State/territory completed studies in*	
New South Wales	46 (31)
Victoria	35 (23)
Australian Capital Territory	12 (8)
Queensland	38 (25)
South Australia	1 (1)
Western Australia	11 (7)
Tasmania	6 (4)
Location not identified	1 (1)
*Academic Status*	
1st year graduate	60 (40)
2nd year graduate	56 (37)
Enrolled in final year of Nutrition &	32 (21)
Dietetics university program	
Academic status not identified	2 (1)
*Encountered a patient with an ED to date?*	
Yes	107 (71)
No	43 (29)
TOTAL	150 (100)

A cross-tabulation of variables (ED type and treatment approaches) as a function of the relevant outcomes (perceived confidence and training needs) is shown in Table 2. Most participants (81% ± 3%) reported lacking confidence in treating patients with an ED, with 96% ± 1% of participants reporting the need for further education and training (Table 2).

**Table 2:** Perceived confidence and need for further training, in treating patients with an ED and engagement in treatment approaches

	Reported Confidence		Requires Further Training	
	Confident %	Not Confident %	Yes %	No %
*Diagnosis*				
Anorexia Nervosa	16	84	95	5
Bulimia Nervosa	16	84	95	5
Binge Eating Disorder	23	77	95	5
ARFID	19	81	97	3
Any ED	19	81	96	4
*Using Treatment Approaches*				
Dietary Counselling	44	56	92	8
Mindful Eating	35	65	93	7
Health at Every Size	29	71	89	11
Intuitive Eating	39	61	91	9
Motivational Interviewing	41	59	95	5
Developing Meal Plans for weight restoration	57	43	85	15
The Real Food Guide	23	77	94	6

ED, eating disorder; ARFID, avoidant restrictive food intake disorder

The most preferred methods to complete further training in the management of EDs were online webinars (27%), followed by workshops (25%); online courses (19%); clinical supervision (15%); site visits to ED services (5%); DA Eating Disorder Interest Group (dietitians who are members of the professional organisation DA, who wish to network with other members in the area of EDs) [45] resources (4%); other/unspecified (3%); and Practice-based Evidence in Nutrition (innovative knowledge translation tool developed by Dietitians of Canada) [46] (2%).

Participants who had previously encountered a patient with an ED were more likely to be confident in providing dietetic services in the area of EDs (Table 3).

**Table 3:** Relationship between exposure to a patient with an ED and confidence to provide dietetic services

		Encountered a patient with an ED			
Confidence to Treat		Yes	No	OR	95% CI
Anorexia Nervosa	Confident	41	5	4.7	1.72, 12.97
	Not Confident	66	38		
Bulimia Nervosa	Confident	35	4	4.7	1.57, 14.32
	Not Confident	72	39		
Binge Eating Disorder	Confident	43	6	4.1	1.61, 10.66
	Not Confident	64	37		
ARFID	Confident	34	5	3.5	1.28, 9.79
	Not Confident	73	38		

ARFID, avoidant restrictive food intake disorder

On the topic of clinical support, 62% (*n* = 93) of participants reported they felt confident knowing where to seek assistance (Table 4). Key sources of support were identified as “other Dietitians” (including clinical supervisor/mentor, ED specialist dietitian, senior dietitian/manager) and evidence based guidelines.

**Table 4:** Main sources of assistance that dietitians use in managing patients with an ED

Source for Assistance	Responses n (%)
Other Dietitians	55 (37)
Evidence Based Guidelines	24 (16)
ED Associations	10 (7)
DA/Interest Group/Resources	10 (7)
MDT Staff	10 (7)
Other Online Resources/Books	8 (5)
ED Services	7 (5)
Webinar/Workshop	3 (2)
Total No. Responses	127 (85)

ED, Eating Disorder; DA, Dietitians Australia; MDT, Multidisciplinary Team

## Discussion

The present study provides insight into the educational and professional development needs of student dietitians and new graduate dietitians in the area of EDs, and reports on their perceived confidence to practice professionally in treating EDs. Currently, dietitians may be more likely to encounter a patient with an ED within the community setting, with considerable growth in the number of new graduate dietitians’ in Australia working in private practice (24%) compared to more traditional settings, such as public hospitals (23%) [17]. The introduction of government incentives in Australia through the MBS to support ED service provision in the community in 2019 [16], may further drive this trend in the future. Practically this means that dietitians may be more likely to encounter both diagnosed and undiagnosed ED patients in private practice clinics [47], with many patients expected to seek treatment of symptoms even in the absence of a formal ED diagnosis. Hence, the traditional views that ED training should be reserved for experienced or specialist dietitians seems redundant in the current context [47].

Our survey findings indicate final year student and new graduate dietitians perceive a lack of confidence to practice confidently in the area of EDs and they identify a number of training gaps. This result was expected given that experienced dietitians also report a lack of professional confidence in working with clients experiencing mental health issues [48]. Future research examining the ways new graduate dietitians plan to upskill in this clinical area would be valuable, and importantly, whether these activities are likely to positively influence their practice should be examined. Growing professional confidence when working within EDs is paramount, for not only the health care professional, but their patients. Greater recognition of symptoms, referral and treatment, as well as a reduction in stigmatised beliefs, may improve with further training [32].

Encounters with patients with EDs vary between health professionals and a lack of exposure to these patients may affect confidence to practice. Physicians typically report minimal encounters [49]. In contrast, findings of the present study suggest that responding new graduate dietitians and final year students on clinical placement, had a reasonably high number of ED encounters (71% of respondents), but due to low response rate the generalisability of the findings are limited. While this higher proportion may be explained by self-selection bias (that is, dietitians and final year students with an interest in EDs who agreed to participate in the survey), dietitians may also be more likely than physicians to encounter patients with an ED as food is a core aspect of an ED.

Clinical placement experiences are integral to workforce preparation, as practical experiences have been shown to influence confidence levels and they have the potential to shape career path intentions [50]. Despite this, current Australian accredited dietetics programs have limited ED placement opportunities, influenced largely by placement partner collaboration agreements that have a small number of ED offerings, and geographical allocation of students [29, 51]. This appears to also be a barrier in other countries and across different disciplines such as medicine, where from 637 residency programs in the USA, only 42 formal ED training rotations were offered [27]. This situation is unlikely to change, hence alternatives that do not require more clinical placement capacity are important to consider. For example, student participation in ED-specific simulations or objective structured clinical exams may offer greater pragmatic training opportunities, without increasing the burden on placements. These could be developed through collaboration between universities and dietitian advocacy groups, such as the DA Eating Disorder Interest Group. Once fully qualified, continual professional development becomes integral to the ongoing learning development of dietitians, in order to maintain and enhance the knowledge and skills they need to deliver a professional standard of service. Mentorship and clinical supervision were identified by respondents in the present study as important professional development engagements.

Evidence based guidelines, including treatment manuals [52, 53], are an important part of navigating uncertainty in clinical practice. Prior to the recent publication of the ANZAED practice and training standards for dietitians [23], and the work of McMasters et al. (2020) on the role of the dietitian and nutrition in evidence based-treatment [24, 53, 54], there was a lack of guidance for dietetic practice in EDs. This may have contributed to dietitians’ perceived lack of confidence in ED treatment. For example, The Royal Australian and New Zealand College of Psychiatrists clinical practice guidelines [2], refer to dietitians’ involvement but they lack specific recommendations for implementation in daily practice. In the UK, the National Institute for Health and Care Excellence Guidelines [55] do not specify that nutrition psychoeducation and management of refeeding syndrome should be completed by a dietitian, while the American Psychological Association [56] stipulates that dietitians may only provide structured meal plans to ensure nutritional adequacy.

Although dietitians recognise the importance of facilitating behaviour change in managing patients with EDs, often they lack knowledge of specific behaviour change methods to effectively support and care for people with EDs [57]. Our survey findings indicated more than half of final year student and new graduate dietitians lacked confidence in applying treatment approaches such as Motivational Interviewing. Insights into mental health concerns and communication skills are fundamental to dietetic practice generally, and specifically in relation to EDs, an understanding of cognitive behavioural therapy principles is required [36, 58]. Acquiring these skills could lead to improved confidence in identification and initiation of conversations with a patient, promoting timely specialised treatment [47] and facilitation of change in eating behaviours [29].

Some limitations were encountered during this research, notably self-selection bias of participants who may have had a particular interest in EDs and greater exposure to them, potentially resulting in divergent perceptions to the broader dietetics workforce. The response rate was low and some states where participants completed their studies were over represented, which limits the generalisability of the findings to the broader dietetic community. Also, incomplete survey responses were not further explored. The low response rate suggests that relying solely on a professional organisation representing dietitians in Australia for survey distribution did not capture a high level of interest in participation. Approaching and engaging specific ED organisations and advocacy groups for a more targeted approach in survey distribution, may have resulted in a higher response rate and should be considered in future studies.

Further research evaluating current course curricula through perspectives of course convenors would be valuable. Exploring perspectives of dietitians with longer term and more diverse practice histories, is also recommended to provide deeper insights into issues highlighted in this study. A better understanding of workforce preparation for graduate dietitians specifically is also warranted in future studies, as is knowledge of current professional settings and private practice experiences. This collective knowledge would assist in identifying potential barriers and enablers in meeting the growing dietetic demands for ED services [59].

## Conclusions

In conclusion, findings of this survey confirm there is an overall lack of confidence in final year student and new graduate dietitians to work with patients with an ED. A need for further ED-specific education and professional development was highlighted. Widely accessible tertiary and post graduate training opportunities in counselling skills (e.g. motivational interviewing), as well as participating in structured clinical supervision may improve practitioner confidence and improve patient outcomes.

## Data Availability

The datasets used and/or analysed during the current study are available from the corresponding author on reasonable request.

## Abbreviations

ED: Eating Disorder
ARFID: Avoidant/restrictive food intake disorder
MBS: Medicare Benefits Schedule
NCSD: National Competency Standards for Dietitians
NEDC: National Eating Disorder Collaboration
DA: Dietitians Australia
MDT: Multidisciplinary Team
ANZAED: Australian and New Zealand Academy for Eating Disorders
